# INVS Mutation-Related NPHP2 Nephronophthisis With Glomerulocystic Disease: A Case Report

**DOI:** 10.1016/j.xkme.2024.100956

**Published:** 2024-12-27

**Authors:** Yuichiro Sawada, Akinari Sekine, Yuki Oba, Masayuki Yamanouchi, Tatsuya Suwabe, Kei Kono, Keiichi Kinowaki, Kenichi Ohashi, Yutaka Yamaguchi, Takuya Fujimaru, Takayasu Mori, Eisei Sohara, Shinichi Uchida, Takehiko Wada, Naoki Sawa, Yoshifumi Ubara

**Affiliations:** 1 Nephrology Center and the Okinaka Memorial Institute for Medical Research, Toranomon Hospital, Japan; 2 Department of Pathology, Toranomon Hospital, Japan; 3 Department of Pathology, Graduate School of Medical and Dental Sciences, Tokyo Medical and Dental University Tokoyo, Japan; 4 Yamaguchi’s Pathology Laboratory, Chiba, Japan; 5 Department of Nephrology, Graduate School of Medical and Dental Sciences, Tokyo Medical and Dental University, Tokoyo, Japan

**Keywords:** Nephronophthisis (NPHP) type 2

## Abstract

We examined a 68-year-old woman with decreased renal function (serum creatinine level of 1.77 mg/dL) and polycystic kidney disease. Magnetic resonance imaging revealed multiple bilateral renal cysts with uniform low intensity on T1-weighted images and uniform high intensity on T2-weighted ones but no mixed intensity cysts. Kidney biopsy findings included collapsed glomerular structures within dilated cyst-like structures. Glomerulocystic disease was diagnosed. Genetic analysis revealed 2 different INVS (nephronophthisis 2 [NPHP2]) compound heterozygous missense mutations. The NPHP2 is usually found in infants, and to our knowledge, this is the first report of an older patient with NPHP2.

Nephronophthisis (NPHP) is an autosomal recessive condition characterized by chronic tubulointerstitial disease with renal cystic lesions and progression to end-stage renal disease (ESRD). Cases of NPHP are broadly classified into the following 3 types: infantile NPHP, which becomes ESRD at around 1 year of age and is associated with NPHP2; juvenile ESRD, which develops at around age 13 and is associated with NPHP1; and adolescent ESRD, which develops at around age 19 and is associated with NPHP3.^1^, ^2^, ^3^, ^4^, ^5^ The NPHP2 associated with infantile NPHP resides on chromosome 9q21–q22.The kidney phenotype of NPHP2 combines features of NPHP, including tubular basement membrane disruption and renal interstitial fibrosis, with features of polycystic kidney disease (PKD), including enlarged kidneys and widespread cyst development.^2^ The renal diagnostic imaging features of NPHP have been summarized as multiple cystic lesions at the cortical medullary junction,^3^^,^^5^ and the renal histologic features, as cystic dilatation of tubules.^3^^,^^4^

We performed genetic testing on a 68-year-old woman who had been diagnosed with cystic kidney disease. We were surprised to find evidence of a compound heterozygous missense mutation in INVS (NPHP2) and even more surprised when a kidney biopsy revealed glomerulocystic disease. We present the details of this case below.

### Case Report

A 68-year-old woman with cystic kidney disease visited our hospital with decreased renal function and hypertension. The patient’s parents had no obvious cystic disease.

On admission, the patient was 157.0 cm tall and weighed 49.0 kg. Her blood pressure was 129/94 mm Hg under antihypertensive medication and temperature was 36.6 °C. Heart and breath sounds were normal, and edema was present in the bilateral lower extremities.

Laboratory findings were as follows: erythrocytes, 3.7 × 10^6^/μL; hemoglobin, 11.8 g/dL; leucocytes, 6000/μL; thrombocytes, 17.2 × 10^4^/μL; total protein, 6.7 g/dL; serum albumin, 3.9 g/dL; urea nitrogen, 37 mg/dL; serum creatinine, 1.77 mg/dL; uric acid, 7.3 mg/dL; estimated glomerular filtration rate, 22.9.1mL/min/1.73 m^2^; sodium, 142 mmol/L; potassium, 5.2 mEq/L; chloride, 111 mmol/L; serum calcium, 8.8 mg/dL; phosphate, 4.6 mg/dL; glucose, 89 mg/dL; hemoglobin A1c, 5.8%; C-reactive protein, 0.1 mg/dL; immunoglobulin (Ig) G, 1,298 mg/dL; IgA, 211 mg/dL; IgM, 61.1 mg/dL; and CH50, 57 U/mL (normal value, > 30 U/mL). The urinary protein excretion was 0.27 g/day and the urinary sediment contained less than 1 erythrocyte per high-power field.

Computed tomography (CT) showed numerous cysts in both kidneys but no cysts in the liver. The total kidney volume of both kidneys was 636 cm^3^ with the right kidney of 344 cm^3^ (12.7 cm × 6.7 cm × 6.5 cm) and the left kidney of 291 cm^3^ (13.8 cm × 6.4 cm × 6.2 cm) (calculated from CT images by synapse Vincent software).^6^ On magnetic resonance imaging (MRI), most of the cysts appeared as homogeneous low-intensity areas on T1-weighted images and as homogeneous high-intensity ones on T2-weighted images (Fig 1). The patient has 1 younger brother who does not have the same disease. Neither parent has the same disease. A kidney biopsy was immediately performed.

**Figure 1:**
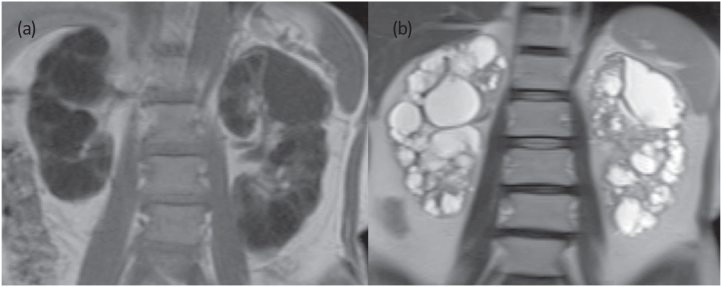
Magnetic resonance imaging of the kidney (coronal cross-sectional view). (A) T1-weighted image showing multiple cysts with uniform low intensity (arrow) in both kidneys and (B) T2-weighted image showing multiple cysts with uniform high intensity (arrow) in both kidneys.

#### Kidney Biopsy Findings

On light microscopy, 4 of the 10 glomeruli sampled showed global sclerosis. Most of the remaining glomeruli were collapsed with dilatation of Bowman’s capsule (Figs 2A and B). Some glomeruli showed irregular dilatation of Bowman’s capsule (Fig 2C). Some cysts were glomerular cysts with flattened parietal cells. No obvious tubular dilatation was seen. Interlobular arteries with thickened walls were also noted. An immunofluorescent study was negative for IgG, IgA, IgM, and C3. Electron microscopy showed no significant electron-dense deposits, but did show a tortuous, collapsed glomerular structure with mild subendothelial edema (Fig 2D). Glomerulocystic disease was diagnosed.

**Figure 2:**
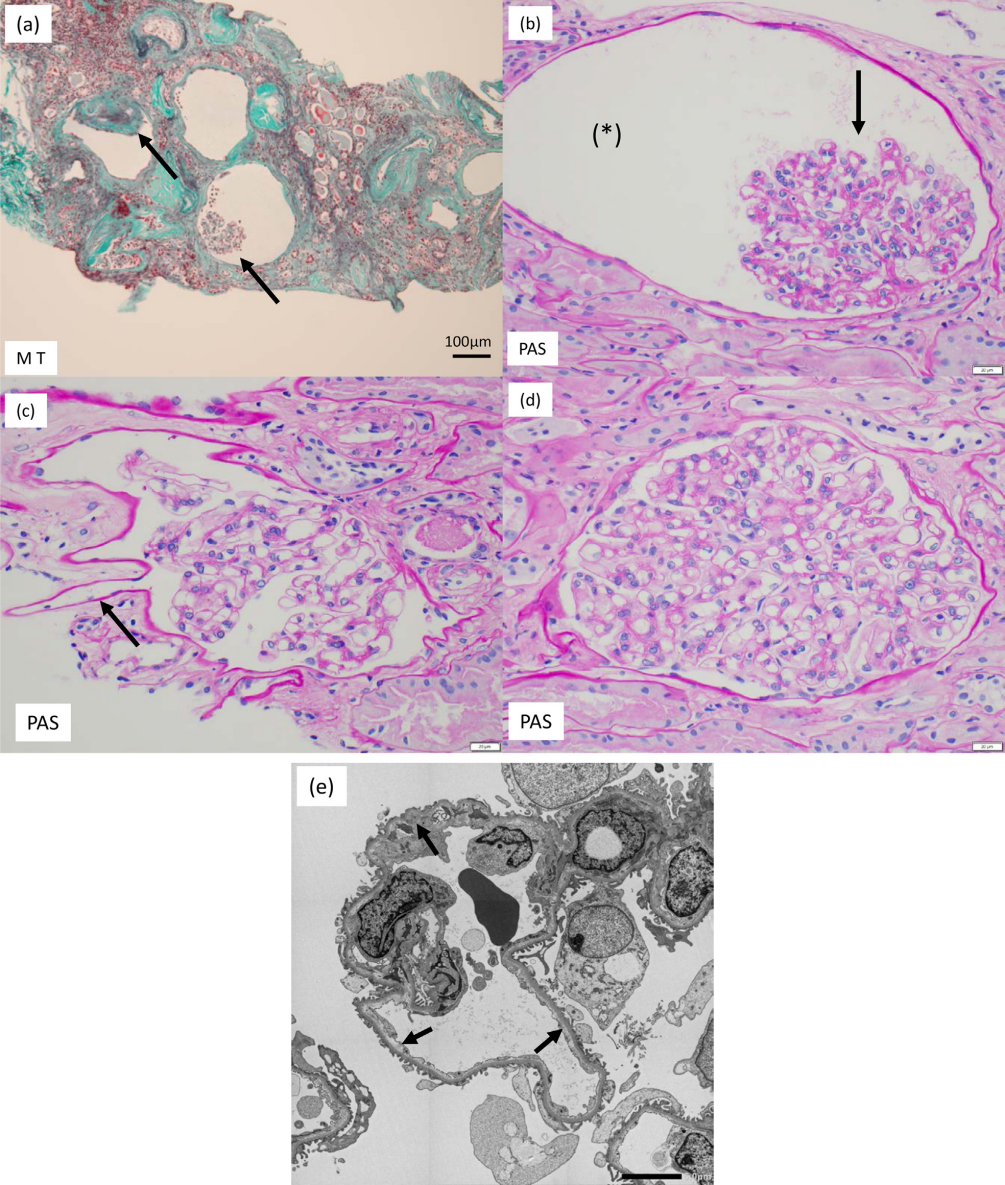
Kidney biopsy findings. (A-D): light microscopy findings. (A) Masson trichrome stain (original magnification, ×100) and (B-D) periodic acid-Schiff stain (original magnification, ×400). (B) collapsed glomerular structures (arrow) were seen within the dilated cyst-like structures (∗); (C) one Bowman’s capsule had a starfish-shaped structure; (D) glomeruli with preserved morphology were enlarged to a maximum diameter of 270 μm; (E) electron microscopy findings. The glomerular structure was tortuous and collapsed, with mild subendothelial edema (arrow) (original magnification, ×1500).

#### Genetic Testing

The following panel of 70 cystic kidney disease genes were searched: AHI1, ANKS6, AQP11, ARL13B, ARL6, ASS1, B9D1, B9D2, BBIP1, BBS1, BBS2, BBS4, BBS5, BBS7, BBS9, BBS10, BBS12, C5orf42,CC2D2A, CCDC28B, CEP41, CEP83, CEP164, CEP290, CSPP1, DCDC2, GLIS2, HNF1B, IFT27, IFT43, IFT122, IFT140, IFT172, INPP5E, INVS, IQCB1, KIF7, MKKS, MKS1, MUC1, NEK8, NOTCH2, NPHP1, NPHP3, NPHP4, orofaciodigital syndrome type 1 (OFD1), PDE6D, PKD1, PKD2, PKHD1, RPGRIP1L,SDCCAG8, SLC41A1, TCTN1, TCTN2, TCTN3, TMEM67, TMEM138, TMEM216, TMEM231, TMEM237, TRIM32, TTC8, TTC21B, UMOD, and WDPCP,

Genetic testing showed the following compound heterozygous missense mutations in INVS (NPHP2): INVS (NM_014425.3): c.1390G>A: p.Ala464Thr and INVS (NM_014425.3): c.1943A>G: p.Asn648Ser.

## Discussion

The NPHP2-associated NPHP is the infantile form and has been reported to progress to end-stage renal disease at around 1 year of age; however, in the present case the disease manifested when the patient was in her 60s. To our knowledge, this is the first case of NPHP2-associated NPHP first appearing in an older adult. We also believe that this is the first report in which the cystic lesions in NPHP2-associated NPHP were glomerular cysts.

Various groups have published ultrasound and MRI findings in NPHP. Aguilera et al wrote that in NPHP, ultrasound shows increased echogenicity with loss of corticomedullary differentiation or the presence of corticomedullary cystic lesions.^7^ Regarding MRI findings, Stafrace et al found that MRI reveals small renal cysts, mostly at the corticomedullary junction and in the medulla,^8^ and Takada et al reported that in a patient with NPHP4, it showed replacement of both kidneys by cystic lesions with low signal intensity on T1-weighted images and high signal intensity on T2-weighted images and that absence of definite cyst walls was a characteristic finding.^3^

Various renal tissue lesions have been described in NPHP. Takada et al reported that dilated tubular lesions, mainly in the distal loop of Henle and the distal tubules, are characteristic of cystic lesions of the kidney.^3^ On the contrary, Fujimaru et al wrote that thick tubular basement membrane duplication is a characteristic feature of NPHP.^4^ Autosomal dominant polycystic kidney disease (ADPKD) is usually a cystic disease of tubular origin, although in rare cases, glomerulocystic disease may also be present. Glomerular cystic disease includes the following diseases: tuberous sclerosis complex, medullary cystic kidney disease type 2, familial juvenile hyperuricemic nephropathy, and OFD1. Currently, only the adolescent form of NPHP, which is associated with the NPHP3 gene, is associated with glomerulocystic disease.^9^ Iijima et al were the first to report that cyst formation in patients diagnosed with OFD1 was of glomerular origin,^10^ and in another article, they reported that in patients with OFD1, renal cysts were enlarged to the same degree as in patients with ADPKD.^11^

Our patient had compound heterozygous missense mutations in INVS (NPHP2). A case report with a compound heterozygosity similar to our genetic mutation was published by Tang et al, who described a 4-year-old girl with estimated glomerular filtration rate of 47.2 mL/min/1.73m^2^. The genetic test showed compound heterozygous mutations of c.2686G>A (p.896V>I) in exon 14 (paternal) and c.1943A>G (p.648N>S) in exon 13 (maternal) of the NPHP2/INVS gene.^12^ However, no histological examination of the kidneys was performed. Although the 2 patients had similar genetic mutations, we could not find any clinical similarities because of the large age difference between them.

Our case differs from ADPKD in the age group of 60 years, which is a typical example of cystic kidney disease, in the following ways: the liver cysts are absent in our case, the total kidney volume of the kidney is smaller, the size of each cyst is smaller, and each cyst has equal intensity on MRI.

The question of the indication for kidney biopsy in cystic kidney disease is answered as follows. In Japan, kidney biopsies have been performed on patients with low urinary protein levels and impaired renal function for which no cause could be identified. This patient’s kidneys were not large despite the presence of numerous cysts. A kidney biopsy was performed to determine the cause of the decreased renal function.^13^

In summary, although NPHP2-associated NPHP usually occurs in infants, we experienced a case in a woman in her 60s. Furthermore, to our knowledge, this is the first time that glomerulocystic disease has been reported in an adult with NPHP2-associated NPHP.

### Limitations

Both of the 2 INVS variants recognized in this study correspond to variant of unknown significance when evaluated according to the American College of Medical Genetics guidelines.^14^ Therefore, it may not be possible to determine that this variant is the responsible gene in this case. The search should include genetic analysis not only of the patient but also of the parents. However, in this case because only the patient's own analysis was available, it may not be possible to determine that the 2 variants are compound heterozygous mutations in different alleles.
